# Understanding how education/support groups help lone mothers

**DOI:** 10.1186/1471-2458-10-4

**Published:** 2010-01-04

**Authors:** Ellen L Lipman, Meghan Kenny, Susan Jack, Ruth Cameron, Margaret Secord, Carolyn Byrne

**Affiliations:** 1 Department of Psychiatry and Behavioural Neurosciences, McMaster University, 1200 Main St. W., Hamilton, Ontario, L8N 3Z5, Canada; 2 Offord Centre for Child Studies, Patterson 206, Chedoke Site, 566 Sanatorium Road, Hamilton, Ontario, L9C 1Y3, Canada; 3 Department of Nursing, McMaster University, 1200 Main Street West, Hamilton, Ontario, L8N 3Z5, Canada; 4 Faculty of Health Sciences, University of Ontario Institute of Technology, 2000 Simcoe, St. North, Oshawa, Ontario, L1H 7K4, Canada

## Abstract

**Background:**

Lone-mother led families are at increased risk of psychosocial disadvantage, social isolation and mental health morbidity. Community-based programs are more accessible for families seeking assistance. We examine the experiences of eight lone mothers participating in a larger randomized controlled trial (RCT) of a community-based education/support group program using mixed methods.

**Methods:**

A purposeful sample of eight mothers participating in the intervention arm of an RCT of community-based support/education groups was selected for the qualitative study. Individual interviews asked mothers about themselves and their relationships with their children before and after the group. Interviews were taped, transcribed and content analysis was used to code and interpret the data. Quantitative data collected in the RCT were used to describe these mothers.

**Results:**

Mothers participating in the RCT and qualitative study experienced multiple difficulties, including financial and mood problems. These mothers reported that before participating in the group, they had shared experiences of social isolation, stigma, a sense of failure, poor relationships with their children and difficulties with financial management. After the group, mothers identified improved self-esteem, support from other mothers, improved parenting skills and improved communication with their children as outcomes of group participation.

**Conclusions:**

The qualitative data revealed mothers' perceptions of specific areas that improved by participating in the group. The utility of complementary information provided by qualitative and quantitative methods in understanding program impact, as well as the need for broader assistance is noted.

## Background

Lone mother-led families make up over one in eight Canadian families (12.7%), and constitute over one million families (1,065,365) [1]. These mothers, classified by the Canadian Census as not legally married or living common-law, often have low levels of education and their families face economic stresses [2,3], as well as social isolation, and health difficulties. For example, lone mothers endorse higher levels of depressed mood and family stress as well as lower levels of social support compared with mothers from two-parent families [4-8]. Children from lone-mother families have elevated rates of emotional and behavioural problems, and academic and social difficulties compared with children from two-parent families[5,7]. Children from poor lone mother families have higher rates of these difficulties than children from non-poor lone mother families or poor or non-poor two-parent families[7].

There are a number of obstacles in the delivery of health and other services to these mothers and children, including availability of services, and barriers to service use such as cost, location/transportation, stigma, childcare, language differences, cultural concerns and literacy [9,10]. Existing traditional mental health services are too few in number to meet the complex needs of these mothers.

With a view to providing service for this multiply disadvantaged population, we ran a community-based group program of social support and education for lone mothers. Prior to this community-based version, the group program had shown promise in an uncontrolled evaluation of lone mothers attending a outpatient child mental health clinic with their children[11]. The placement of the trial in the community provided an opportunity for increased access to families who often face substantial barriers[9]. The objective of this randomized controlled trial was to assess the effect of group participation on self-reported well-being (mood, self-esteem, social support) and parenting. We demonstrated that mothers randomized to group participation had some positive short-term effects on mood and self-esteem but no differential impact on social support or parenting[12]. Both intervention and control groups showed improvements on all measures of functioning on longer-term follow-up, with no statistically significant differences between them. Participant ratings of group process, specifically group cohesion, were also strong, and significant associations were found between group cohesion and specific positive outcomes[13].

The use of quantitative changes on scale scores provides useful information about an individual's change on an outcome of interest, and about treatment effects. Based on our quantitative analyses, there are at least short-term improvements associated with group participation. Further understanding of how and why participants improve or what prevents improvement can be gained through qualitative inquiry. Increasing our awareness of the values, meanings and preferences of the participating mothers enables identification of shared and unique processes experienced by participants, and opportunities for program improvement and supplementation. We added a qualitative component to our study of high-risk mothers to further understand the benefits and limitations of our community-based group program.

The objectives of this paper are to: (i) describe a sample of eight mothers participating in the education/support group trial and qualitative interviews, and (ii) present the results of qualitative interviews in an effort to further understand the impact of group participation on maternal well-being including social support and self-esteem, parenting and the parent-child relationship for these mothers.

## Methods

The overall project was a concurrent, embedded mixed methods study where a small qualitative study was embedded within a larger randomized controlled trial (RCT). For the embedded qualitative study, principles of fundamental qualitative description [14]were used to guide the sampling, data collection and analysis processes. This type of qualitative approach is used to provide a comprehensive summary of facts and events, using the 'everyday' language of the participants, and is commonly used by researchers who require answers to questions about specific events or phenomena[14].

### Participants

Details of the RCT have been published elsewhere[12], and will be described briefly. The RCT was approved by the Research Ethics Board of Hamilton Health Sciences/Faculty of Health Sciences, McMaster University (REB #99-216). Lone mothers with young children were recruited through advertisements in community flyers in Hamilton, Ontario, asking if mothers were "feeling alone" or "parenting on their own." Mothers classified themselves and the study team did not check or exclude mothers who classified themselves as lone mothers but had a partner living in the home. Inclusion criteria were (1) had at least one child 3-9 years of age, (2) spoke English, (3) had no acute psychiatric crisis (e.g., suicidal behaviour) or threat of violence (e.g., by ex-partner), and (4) provided informed written consent to participate in the trial. One hundred and seventeen (117) mothers were eligible and agreed to participate in the study. Participants completed baseline questionnaires then were randomly assigned to either an intervention condition (59) or control condition (57).

The subsample that consented to participate in the embedded qualitative study was eight mothers who were randomized to the intervention group. A purposeful sampling approach was taken with the intention of yielding information-rich cases that could provide insight into the participants' experiences in the program. Information-rich cases allow the researcher to glean information of particular salience to the focus of the research[15]. Although a sample of convenience, this sample was also purposeful in that the participants were selected for qualitative interviews based on (i) having participated in the intervention group, (ii) completed pre- group, post-group and at least one further follow-up interview at 12- and/or 18-months, (iii) had up-to-date contact information, and (iv) expected to be agreeable to an additional in-home qualitative interview. All mothers who were approached agreed to participate. No other mothers were included due to budgetary limitations. Mothers were given $25.00 retail certificates for participating in the qualitative interviews.

### Intervention

Details of the intervention have been published elsewhere[12], and will be described briefly. Mothers assigned to the intervention group were asked to participate in a 10 week, 1.5 hour per week group program that provided social support and education at a community location. Each support group was made up of 6 to 10 women and two trained leaders. A manualized group program was used, with content covering two main areas: child themes (e.g., normal and deviant development and behavior, behavior management, child welfare) and maternal themes (e.g., social isolation, financial stress, coping, relationships). Children three to nine years old could attend an activity-based group program that ran concurrently with the mothers' group sessions. Mothers in the intervention arm received weekly phone reminders and transportation assistance (bus tickets, gas/parking money, or taxi fare).

### Data Collection

Quantitative assessment data were collected at baseline, post-group, and 12 and 18-month follow-up from all participants (intervention and control) by paired interviewers at home visits. At each visit, mothers and children received gifts of appreciation for their participation (e.g., retail gift certificates, children's books).

In-depth, semi-structured interviews were completed with the subsample of eight mothers recruited from the intervention group (see participant section above). An interview guide was developed (Additional File 1), with the main questions focusing on the mothers themselves and on the relationships between mother and child at two time points, before the group and after. For example, mothers were asked to "think back before the group...about how things were (for you) as a single mother." Specific follow-up probes were used to ask about social support, normalization, educational development and resource use, as needed. Mothers were also asked about the importance of continued contact after the group, how they would describe the group to someone else, and to make any additional comments regarding the effects of participation in the group on themselves or on the mother-child relationship. All of the interviews took place in the mothers' homes, were conducted by one of two interviewers, and each lasted 1 to 2 hours. Interviews were audiotaped and transcribed verbatim. Interviewers also maintained field notes over the course of data collection. Both interviewers were female, held masters degrees (M.A.) and had extensive experience conducting qualitative interviews.

### Descriptive Measures

#### Socio-demographic variables

These variables included maternal age (years), history of treatment for a nervous condition in the last 6 months (yes/no), education (highest grade/level completed), employment (worked at a job or business anytime in the past year), income, financial pressure (feels "money is a struggle", yes/no), and sources of financial support over the past year.

Low mood was assessed by the Center for Epidemiologic Studies Depression Scale [CES-D] [16], a 20-item self-report measure of psychological distress, including cognitive, affective and behavioural "state" of depression and respective frequencies. Scores range from 0-60, with higher scores indicating more severe symptomatology. Internal consistency = .84 -.90 [16]. The CES-D has been extensively validated[17].

Self-esteem was assessed by the Rosenberg Self-Esteem Scale[18], a 10-item self-report of self-esteem or psychological coping. Scores range from 10-40, with higher scores indicating higher global self-esteem. Internal consistency ratings range from .72 to .87[19].

Social support was measured by the Social Provisions Scale[20], a 24-item self-report measure of perceived social support (6 subscales: attachment, social integration, reassurance of worth, reliable alliance, guidance, opportunity for nurturance and total). Total scores range from 24-96. Internal consistency = .65 to .76[20]. We use the total score.

Parenting was measured by the Parenting Scale[21], self-report measure of dysfunctional discipline practices in parents with young children. This is a 30-item scale, and total scores range from 30-210. We use the total score of the three subscales (laxness, over-reactivity and verbosity) (alpha = .84)[21].

### Analyses

Quantitative data were analyzed using SPSS version 12[22]. Means and variances for selected descriptive variables were calculated.

In the qualitative analyses, a conventional content analysis approach was used in categorizing interview data. The main benefit of the conventional approach is allowing the researcher to draw information directly from the participants while refraining from applying any theoretical assumptions or predetermined inferences about the data[23]. While referring to their notes, interviewers reviewed all of the transcripts to ensure the accuracy of the transcription. Analysis of the data commenced with examining the interview transcripts and the interviewer notes. Preliminary codes emerging from the data were identified, using the interview guides and the evaluation questions to keep the context of the data in mind. Following this brief overview, phrases were highlighted in the transcripts and viewed in light of the corresponding category, while all examples of a particular category were grouped together. Finally, all of the categories were listed and examined in terms of more broad and overarching themes.

## Results

The objectives of this paper were to: (i) describe a sample of eight mothers participating in the education/support group trial and qualitative interviews, and (ii) present the results of qualitative interviews in an effort to further understand the impact of group participation on maternal well-being including social support and self-esteem, parenting and the parent-child relationship for these mothers.

### Sample characteristics

Table 1 displays the descriptive characteristics of the eight mothers participating in the education/support group trial and qualitative interviews. Mothers who did qualitative interviews ranged in age from 30.3 to 42.9 years, and most had completed high school (5/8), were employed in the past year (6/8), felt money was a stress (6/8), and received social assistance (6/8) and child support (5/8) as forms of financial support in the past year. Further description of the mothers is included with the quotes presented below. We also compared mothers participating in the qualitative interviews (8) with mothers in the intervention group who were not part of the qualitative study (51) (data not shown). There were no significant differences on any of the descriptive characteristics measured except higher social support than the other intervention mothers.

**Table 1:** Baseline characteristics of mothers participating in support/education group trial and qualitative interviews (N = 8)

Characteristic	Mean (SD)	
Age, yr	36.3	(4.6)
	%	
Treated for "nerves" or nervous condition in last 6 mo	75.0	
		
Maternal education		
Some secondary or less	12.5	
Completed secondary	25.0	
Some postsecondary	25.0	
Completed postsecondary	37.5	
		
Employed in past year	75.0	
		
Income <$15,000 in past year	50.0	
Financial pressure	75.0	
Sources of financial support in past year		
Wages and salaries	63.0	
Social assistance	75.0	
Other	75.0	
		
Mood ^a^	20.1	(9.4)
Self-esteem ^a^	20.5	(5.3)
Social support ^a^	40.8	(10.6)^b^
Parenting ^a^	105.4	(16.4)

^a^All outcomes were scored to reflect poor functioning

^b ^Between -- group difference Intervention subjects included in qualitative study (n = 8) vs not included (n = 51) (p < .01), data not shown

### Themes

The main conceptual themes that emerged from the interview with mothers are presented in two parts: 1) experiences of being a lone mother before the group intervention; and 2) experiences of being a lone mother after participation in the group intervention. Anonymised brief descriptions of the participants quoted are included.

#### Before the Group Intervention

##### Life as a Single Mother

-Isolation

When asked what their lives as lone mothers were like prior to their participation in the group, all of the participants described intense feelings of isolation. One mother described feeling that

"I was at probably one of the lowest points as a parent. I felt destitute. I found that I felt absolutely alone in the absolute world." (Louise, 41 years old, college-educated employed mother of two)

The rejection by a partner resulted in a specific type of isolation experienced by these mothers and as one participant explained:

"You feel a little bit isolated and I think you know your self-confidence obviously takes a huge hit when the person that you love basically doesn't love you back or your marriage ends." (Betty, 31 years old, high-school educated mother of 2, employed part-time)

Social isolation was intensified by two key experiences. First, most of the women disclosed that they did not receive social, financial or instrumental support from family and friends. A key consequence of this lack of support was that they were rarely able to have time away from their children to socialize with other adults. Second, many of the women disclosed that their connections to their social circles of friends were severed when their marriages ended.

-Stigma

Contributing to feelings of isolation was the perceived stigma associated with being a lone mother. Concern over other people observing or judging them and their children compounded feelings of isolation by discouraging mothers from socializing or spending any extended amount of time in public places. One participant shared how,

"for the first while I just hid. It was like people could see your problems". (Linda, 39 years old, employed mother of one, did not complete high school)

Another woman alluded to feeling as though she was

"under a magnifying glass" as a single mother and that "everybody's watching you because you're labeled a single mom and there is a stigma and it exists". (Louise)

Augmenting feelings of shame associated with their circumstances were preconceived notions of single mothers and the 'kind' of people who generally fall into this category. Several of the participants disclosed that before becoming lone mothers they held stereotypical negative views of single mothers. One mother noted,

"I was probably one of those people that labeled single mothers. You know, like I'd drive by a housing unit and go, 'Oh look at that' because it doesn't look good." (Louise)

Another mother shared that she felt very different from other people when she became a single mother, and asserted,

"I think [that] before I became a single mother I probably was judgmental of single mothers. You know looking back sort of thinking you know, 'like who are they?' Almost 'what have you done wrong to be in that position?"' (Betty)

-Sense of Failure

Compounding feelings of shame among mothers was an overwhelming sense of failure because 1) they perceived that they were unable to make their marriages work, and 2) they were struggling financially to support themselves and their children. As one woman remarked,

"There's a sense of failure that hey, look at me, I'm in my 30's and I'm on my own and these kids and I don't have a career." (Betty)

One participant shared her resistance to reaching out to her family and friends because of the embarrassment she felt around entering into a relationship of which no one had approved, and which she herself had sensed would not work:

"I was hiding absolutely everything. Oh, I was embarrassed. You know why? I married someone that everyone knew it wouldn't work [with] and in my gut I knew it too." (Louise)

##### Parent-Child Relationship

When asked to describe their relationships with their children prior to starting the group, several of the participants explained that the emotional, mental, and financial stresses that they were experiencing negatively impacted their ability to parent successfully. One mother shared that

"It was really stressful and I did find that my stress levels being so high made me not as good a parent. I lost my temper more and I didn't have as much patience." (Lea, 30 years old, employed part-time, mother of two)

From the interviews, the mothers provided several examples that their high stress levels particularly impacted their ability to effectively discipline their children and they resorted to disciplinary practices that may not have been used in less stressful situations. For example, one mother disclosed,

"Spankings were a lot more and yelling and screaming was a lot more. I wanted to know like how do other single mothers do it without screaming their head off and losing it?" (Linda)

In addition to acknowledging the effect that changes in their own behaviour had on the relationship with their children, many of the mothers also described the negative impact that their children's behaviours had on their parenting relationship. Some mothers shared their uncertainty around how to address their children's anger over all of the changes happening in their lives. One participant explained how,

" [the kids] still have anger and it's like if you don't know how to deal with it... it's really hard" and how she "would react instead of you know, trying to take a step back and let them have their anger." (Betty)

Some of the mothers also described a negative change in the home environment as a result of their children's behaviour. One woman shared that,

"it was so bad, because on a daily basis it was like a living hell, [the child's] anger" and "20 or 30 times a day [my daughter would say] 'I hate you, I hate my life, I don't want to live."' (Linda)

-Learning how to budget/adjusting to managing finances for the family

In addition to having to adjust to parenting on their own, many of the mothers had relied on their former partners to manage their finances and as a result, had to quickly learn to budget and manage their bills when their marriages ended. This added responsibility proved to be challenging for many of the women. One woman admitted,

"I hadn't done the budgeting at all before. I just handed over my pay cheques and he looked after everything and thought everything was okay. So that was a really big step for me, I had to go to Family Services and get help for budget counseling because I just didn't have a clue." (Linda)

Due to the drastic decrease in their incomes, most of the women found themselves having to deal with serious financial problems, which in some cases resulted in the loss of the family home. As a result, they were forced to seek out assistance to compensate for the loss of income. Some of the participants talked about the humiliation and shame that they experienced trying to obtain financial support and housing for their family. For instance, many of the women had gone from being home-owners to living in subsidized housing. One mother shared,

"I was on social assistance and my ex-husband wasn't giving me any support at all so that was difficult. It was hard to live on the money that you get plus I had to buy furniture for the kids and apply for housing and deal with something that I've never dealt with before like social workers and things like that." (Margie, 34 years old, college-educated, self-employed mother of two)

Another mother shared how the process of finding housing after her separation was like begging,

"selling [her] soul, telling people what happened to [her], why [she] was in this situation and [how she] needed a home." (Linda)

#### After the Group Intervention

When asked about the outcomes of group participation, mothers shared several positive outcomes that they had experienced as a result of participating in the group.

##### Life as a Single Mother

-Improved Self-Esteem

When asked to explain how life was for them prior to participating in the group, many of the mothers talked about the damage that going through a divorce and dealing with the perceived judgment of others had on their self-esteem. Several of the mothers also shared how they felt that their children's poor behaviour was a reflection of the lack of parenting skills. Participation in the group and connecting with other mothers helped the participants gain a new inner strength and improved their self-image. One participant summarized some of these key positive outcomes of group participation,

"I felt better. I had more confidence. I learned to use my anger...going to group and talking to the other mothers really helped me a lot to have my inner strength and to think, 'Okay I can do this"'. (Margie)

-Support from Other Mothers

All of the group participants disclosed that they had benefited from the advice, encouragement, and overall support from the other women who were dealing with similar challenges. One participant shared how the reassurance of other group members helped her cope with a particularly difficult time:

" [I started the group] at a time before my children went for their first long visit to [see their father] and so it was very helpful to have the other women in the group telling me that it was okay and it was going to be fine and that they would come back." (Betty)

More importantly, all of the women commented on the decreased sense of isolation that they experienced as a result of connecting with other lone mothers.

"With the group they had the same problems with Social Assistance and that and discrimination about being single mothers sit on your butt all day and do nothing kind of thing. They shared their experiences and it helped me a lot to see that everybody has their own problems, and I'm not the only one and I'm not alone, I'm not the only one out there walking down the street with no husband." (Margie)

For other participants, having opportunities to interact with mothers in similar circumstances who could relate to their struggles provided them with a much-needed environment of acceptance. After being cut off from their social circles, feeling judged and ashamed of their circumstances, finally being surrounded by others struggling with the same circumstances allowed these women to share their fears and emotions. One woman shared how she just

"sat and just sobbed and it was like this is what I needed and then I got guidance and the whole group understood. They completely understood." (Louise)

##### Parent-Child Relationship

-Improved Parenting Skills

Many of the women noted improvements that they had seen in their parenting skills. For instance, one mother shared that she was

"more calm and relaxed and better at parenting now than I was [before the group]. Before it was me being all stressed out and now I've gotten over that and everything is calmer." (Lea)

All of the women felt that they had the ability to parent before the group, but there was a problem with the choices that they had made as parents. Another mother noted that

"After the group [and] the education, the way I felt about myself was better so I was able to try and find some parenting skills. I was able to dig deep and they were all there." (Louise)

This increased sense of calm and patience that participants gained from the group allowed them to realize that they had the strength and ability to parent their children effectively.

-Improved Communication with Their Child

Many of the women that we spoke with shared how the stress they were experiencing combined with coping with their child's behavioural problems led to and increased poor communication methods. Participants explained that the skills that they gained from participating in the group encouraged the use of healthier, more effective communication methods with their children. Some of their situations are shared below:

" [I learned] how to deal with a child who is really quite stubborn and how to talk to them and make sure that whatever you are saying they are absorbing it. I think also to consider children not just as children but as people. You don't have to give the orders all the time you can ask "what do you think about it?" Just talk to them as people. I find that children appreciate when you take their own opinion into consideration. I did find it useful in that way, how to interact with my child and to get better results." (Susan, 36 years old, unemployed mother of two)

Another participant explained how participating in the group encouraged her to shift her focus from he daughter's negative behaviour to acknowledging her good behaviour, allowing her to

"to step back and praise her a little more and talk to her a little more." (Linda)

The combination of decreased stress levels and the acquisition of new parenting skills seemed to create the opportunity for mothers to really listen to their children and be more mindful of their needs.

##### Positive Aspects of Group Participation

The most positive aspect of participation in the group sessions was the opportunity to share personal experiences and discuss day-to-day challenges with a group of mothers who fundamentally understood and had lived through similar experiences. One mother indicated that she

"enjoyed sharing with other people and that it was a good forum to realize that people have some of the same problems that you did or some of the same frustrations that you did. That was beneficial." (Deb, 34 years old, employed part-time, mother of one)

For some of the women who were recently divorced, it was encouraging to see others who had been divorced for quite some time, doing well, forming new relationships and supporting themselves and their children. One mother commented that she,

"got to see that most of the women in my group had been through their divorce separation 10 years before, 5 years before...they were fine and many of them had moved on to other things and relationships and it gave me hope that as time went on I would get used to this single life and find other things in my life." (Betty)

##### Suggestions for Improving the Group

Participants suggested that the group could be improved by: increasing the length of each meeting (to allow everyone a longer chance to speak), increasing the number of group meetings, providing an opportunity for follow-up and closure with other group members, and to consider running the group with people living in the same area to create a stronger sense of community.

## Discussion

Many lone mothers are multiply disadvantaged and our eight study participants were similarly disadvantaged in terms of education, employment, income sources and adequacy, and mood. Scores on the CES-D scale greater than 16 are considered indicative of probable clinical depression [16], and baseline levels indicated exceed this threshold in four of our eight participants.

In this study, when asked about their lives as lone mothers before participating in the group, mothers identified a number of shared negative experiences including isolation, stigma, and a sense of failure. Many also felt they were coping poorly in their role as a parent and managing finances. Based on the experiences described by mothers after the group, improvements related to support from other mothers, better self-esteem, better parenting or more confidence in parenting skills, enhanced communication with their child, and feeling that someone understood their situation. The themes emerging are not surprising to many, including lone mothers, their friends and those who work with lone mothers in health care and other venues.

Many of the comments made illustrate Yalom's therapeutic factors in group psychotherapy, such as instillation of hope, universality, imparting information, development of socialization techniques, and interpersonal learning[24]. For example, comments made about support from other mothers illustrate universality (e.g., "...I'm not the only one and I'm not alone..."). Yalom proposed that these factors represent components of the complex therapeutic experience occurring in group psychotherapy, and the interview process allowed us to better capture these experiential elements.

The qualitative research method and comments made by the mothers supplement and augment our quantitative study data. For example, quantitative analyses from our RCT demonstrated that group participation had some positive short-term effects on mood and self-esteem but no statistically differential impact on social support or parenting[12], although both social support and parenting improved from baseline to post-group follow-up among group (and non-group) participants. Both support and parenting emerged as clear themes related to improvement identified by mothers in the interviews completed after the group.

How can we reconcile the positive qualitative results suggesting that the mothers perceived having experienced positive benefits in social support and parenting skills as a result of the intervention when the quantitative results from the larger evaluation fail to show effects in these same areas (as compared to the control group)? This may be because each method of inquiry addresses a different question[25]. In our larger quantitative study we examined the effect of participation in an RCT on maternal well-being (mood, self-esteem, social support) and parenting; in the qualitative study we asked mothers more broadly about themselves and their relationships with their child before and after the group. The themes of benefits of social support and in parenting emerged from the interview content. Outcomes not measured in the quantitative study were part of the qualitative study (e.g., parent-child relationship). As well, the original RCT proposal was powered to detect differences in mood, though not necessarily for social support or parenting. The discrepant results may also be due to poor sensitivity of the quantitative instruments or the fact that the mothers participating in the qualitative study differ from mothers in the overall sample in a select way, not reflected on their quantitative scores. Mixed methods approaches allow utilization of the strengths of each method while addressing methodologic limitations, offer complementary data, and provide a fuller perspective of the impact of group participation on mothers[25,26], though there is on-going debate about this third research paradigm (see critique in[27]).

Approaches to assisting with some of the difficulties identified by mothers go beyond the provision of this support/education group program. Effective programs targeting improvement of specific skills, such as parenting, are available[28,29]. Education about parenting could also be provided more broadly in universal programs[30]. Incorporation into courses provided in the secondary school curriculum may be helpful to enable mothers (and fathers) to feel better prepared for parenthood[31]. One of the issues raised, the social stigma associated with lone mothers, was experienced by participants, both as recipients of disapproval and, in some instances, past promoters of negative stereotypes. Endeavors to eliminate stigma, through efforts at education and inclusion, often require broad and concerted actions [32].

A number of comments made by mothers included suggestions for improving the group. All of these ideas had been previously raised by mothers and group leaders informally (i.e., not as part of this qualitative study) or considered by the research team. For example, logistic (e.g., timing of groups relative to school day and bedtime for young children) and funding issues limited prolonging single group sessions or extending program length. Efforts were made to provide groups composed of mothers from a more localized area (e.g., subsidized housing project), but inadequate enrollment made this impossible. Issues of confidentiality were an important part of each group, and would likely be more difficult with localized groups.

Limitations of this work should be noted. This work is based on a small sample of convenience. However, from a qualitative perspective, the sample was also selected purposefully (mothers who had participated in the RCT intervention group, remained in multiple follow-up data collections, had up-to-date contact information and who were considered by the research team to be likely to agree to participating in the qualitative interviews). Given the substantial homogeneity of the qualitative sample data saturation of all themes was achieved. From a quantitative perspective, these mothers may not represent the views or experience of all mothers who participated in the intervention group of the trial, though they did not differ on pre-trial characteristics, except social support. The original trial was focused on and funded for quantitative evaluation and the small qualitative inquiry was added at a later stage to the methodology.

## Conclusions

Mothers participating in this qualitative study were multiply disadvantaged, including financial and mood difficulties. Before participating in the group, mothers identified a number of shared negative experiences including isolation, stigma, and a sense of failure. Many also felt they were coping poorly in their role as a parent and managing finances. Based on the experiences described by mothers after the group, improvement related to support from other mothers, better self-esteem, better parenting or more confidence in parenting skills, enhanced communication with their child, and feeling that someone understood their situation.

The results of this exploratory qualitative study provided our research group with improved understanding of how the mothers in the study experienced the group and the meanings attributed to these experiences. This information has conceptual utility, allowing service providers of parenting and support groups for lone mothers to better understand the experiences and perceptions of these mothers. As lone mothers have to cope with many acute and chronic stresses, programs to assist these mothers and their families need to address a complex range of health and social issues. Program evaluation that includes both quantitative and qualitative research will provide the most comprehensive understanding of program utility[33].

## Competing interests

The authors declare that they have no competing interests.

## Authors' contributions

EL designed and conducted the study and drafted the manuscript. MK performed data analysis and data interpretation and assisted with manuscript review. SJ assisted with data interpretation and manuscript review. RC and MS assisted with data collection and manuscript review. CB assisted with study conceptualization and manuscript review.

## Pre-publication history

The pre-publication history for this paper can be accessed here:

http://www.biomedcentral.com/1471-2458/10/4/prepub

## Supplementary Material

Additional file 1 **Figure S1**. Interview Guide. Click here for file
