# Supplementary material for: Understanding how education/support groups help lone mothers
Source: BMC Public Health. 2010 Jan 4;10:4. doi: 10.1186/1471-2458-10-4 (PMC2820475; doi:10.1186/1471-2458-10-4)
Supplement: Additional file 1 — Figure S1. Interview Guide. [file 1471-2458-10-4-S1.DOC]

Figure 1

Interview Guide

Introduction to Interview

Thank you for taking time to talk with me. As you know, I am involved in research on the effects of support/education groups for moms who are parenting on their own.

You have taken part in the Alone Mothers Together group (date), and helped us by filling in questionnaires before the group, after the group and on one or two follow-up interviews. Today I will be asking you to tell me about your thoughts and ideas about participating in the group, both positive and negative.

The reason I am taping the interview is so I don’t miss any of your comments. The interview will take one to two hours.

1. Before the Group
2. About you

I would like to start by asking you to think back before the group, and ask you about how things were for you as a single mother.

Probes:

Did you have friends/relatives who you could turn to for support?

Did you feel like you were somehow different from other people since you were parenting on your own?

Did you know what to expect in terms of how your child will grow and develop and behave? (if need to, give example about developmental milestones)

Did you know what to expect in terms of how to be a parent? (if need to, give example about limit setting)

Apart from your friends/relatives, did you have other supports or resources that you used?

1. About you and your child

I would like to ask you to tell me about your relationship with your child before the group.

Probes:

Can you tell me about some positive things about your relationship with your child?

Can you tell me about some difficulties in your relationship with your child?

1. After the Group
2. About you

I would like you now to think about after the group, and to ask you how things were for you then as a single mother.

(Note: when there are differences in responses from before the group questions, ask whether the mom feels this is due to the influence of the group).

Probes:

Did/do you have friends/relatives who you could turn to for support?

Did/do you feel like you were somehow different from other people since you were parenting on your own?

Did/do you know what to expect in terms of how your child will grow and develop and behave?

Did/do you know what to expect in terms of how to be a parent?

Apart from your friends/relatives, did/do you have other supports or resources that you used?

1. About you and your child

I would like to ask you to tell me about your relationship with your child after the group.

Probes:

Can you tell me about some positive things about your relationship with your child?

Can you tell me about some difficulties in your relationship with your child?

3. Some people run groups for moms which just stop at the end, and other groups have some continued contact after the group (like our group does). How important do you think this continued contact is?

Probes:

How would you modify the group to make it better or more useful?

1. If you were asked to describe the mothers’ group to someone else, what would you say?

Probes:

What positive things would you say about the group experience?

What negative things would you say about the group experience?

What differences in your life have taken place because of the group?

1. Are there any additional comments you would like to make regarding the effects of participation in the group on you or on you and your child that we haven’t covered in this interview?

I would like to thank you for your time and for participating in the interview.

…
